# 2440. Central Line-Associated Bloodstream Infection Incidence Reduction Accompanies Initial-Specimen Diversion Device Adoption

**DOI:** 10.1093/ofid/ofad500.2059

**Published:** 2023-11-27

**Authors:** Cody G Stroupe

**Affiliations:** Magnolia Regional Health Center, Corinth, Mississippi

## Abstract

**Background:**

Established lines are poor sites for sampling blood (frequently host to enterococci and *Candida* species) but peripheral venipuncture is an imperfect alternative as antiseptics struggle to eliminate deeply embedded microorganisms. State-of-the-art initial-specimen diversion device (ISDD) use can facilitate near-zero blood culture contamination rates, but impact on central line-associated bloodstream infection (CLABSI) incidence is unclear.

**Methods:**

Intervention took place within a Joint Commission-accredited, 200-bed, acute-care community hospital serving nine Southeastern United States counties toward the objective of reduced CLABSI reporting. From September 2021 through March 2023, ISDD use augmented all peripheral venipuncture sample collection for adult patient blood culture. CLABSI reporting and the associated National Healthcare Safety Network (NHSN) standardized infection ratio (SIR) data for the 18-month intervention period was compared to the preceding 18-month period.

**Results:**

For the 18 months prior to ISDD use, 6.5 CLABSIs were predicted and 17 CLABSIs were reported. For the 18 months during which ISDD use augmented standard procedure, 5.5 CLABSIs were predicted and 3 CLABSIs were reported. ISDD use accompanied an 80% reduction in SIR, from 2.6 to 0.5.

**Figure:**
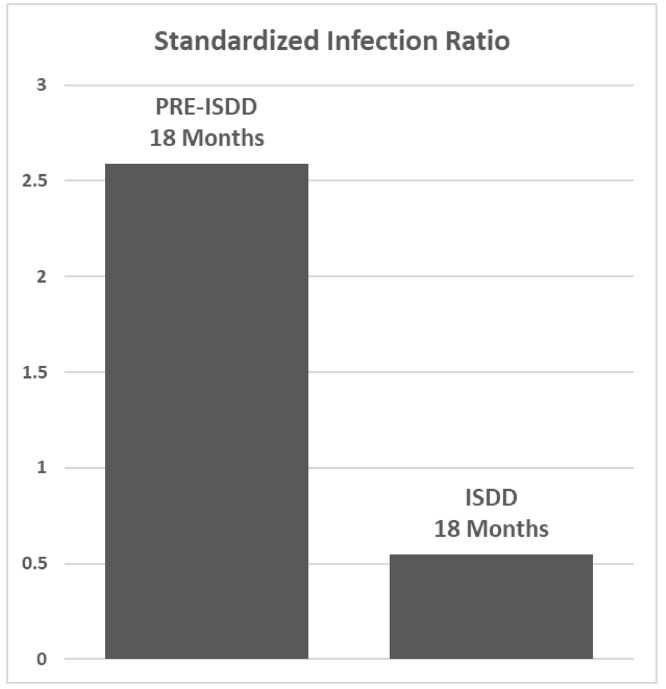

**Conclusion:**

The study period coincided with the COVID-19 pandemic, a nationwide struggle with considerable quality control shortcomings observed in this space that, by juxtaposition, reinforce the strengths of the intervention. SIRs are the primary means of tracking healthcare-associated infection trends, and financial penalties follow performances failing to meet predictions. As CLABSI reporting throughout the intervention period was dramatically below NHSN prediction, the author concludes that ISDDs have standard-of-care potential and encourages further study in the interest of antimicrobial stewardship and quality control.

**Disclosures:**

**All Authors**: No reported disclosures

